# Supplementary material for: Auditory sensory deprivation induced by noise exposure exacerbates cognitive decline in a mouse model of Alzheimer’s disease
Source: eLife. 2021 Oct 26;10:e70908. doi: 10.7554/eLife.70908 (PMC8547960; doi:10.7554/eLife.70908)
Supplement: Supplementary file 1. [file elife-70908-supp1.docx]

**Supplementary Table 1.** List of reagents and antibodies used.

| Reagent type | Designation | Source | Identifiers | Additional information |
| --- | --- | --- | --- | --- |
| Commercial assay | Dihydroethidium (Hydroethidine) DHE | Thermo Fisher Scientific | Cat. No. D23107 | 1μM |
| Mounting medium | FluoSave | Merk Millipore | Cat. No. 345789 |  |
| Antibody | anti-4HNE  (rabbit polyclonal) | Alpha Diagnostic International | Cat. No. #HNE11-S | IF (1:100) |
| Antibody | Anti-rabbit IgG  AlexaFluor 546 | Thermo Fisher Scientific | Cat.No. # A-11035 | IF (1:400) |
| Nuclear Counterstains | DAPI | Thermo Fisher Scientific | Cat.No. D1306 | IF (0.5 mg/mL) |
| Buffer lysis | RIPA Buffer | Sigma-Aldrich | Cat. No. R0278 |  |
| Color standards | Precision Plus Protein Dual Color Standard | BioRad | Cat. No. #1610374 |  |
| Antibody | Anti- Phospho-AMPA Receptor 1 (GluA1, Ser845)  (rabbit polyclonal) | Cell Signaling | Cat. No. #8084 | WB (1:1000) |
| Antibody | Anti-AMPA Receptor 1 (GluA1) (mouse polyclonal) | Millipore | Cat. No. #2263 | WB (1:1000) |
| Antibody | Anti-PSD-95  (rabbit polyclonal) | Cell Signaling | Cat. No. #3450 | WB (1:1000) |
| Antibody | Anti-Phospho Tau Ser396  (rabbit polyclonal) | SAB Signalway Antibody | Cat. No. #11102 | WB (1:1000) |
| Antibody | Anti-Tau-5  (mouse monoclonal) | Thermo Fisher Scientific | Cat. No. MA5-12808 | WB (1mg/mL) |
| Antibody | Anti-TNF-α  (mouse monoclonal) | Santa Cruz Biotechnology | Cat. No. sc-52746 | WB (1:500) |
| Antibody | Anti-Mn-SOD (SOD2)  (rabbit polyclonal) | Merk Millipore | Cat. No. #06-984 | WB (1:1000) |
| Antibody | Anti-Nitro tyrosine  (rabbit polyclonal) | Abcam | Cat. No. ab42789 | WB (1:1000) |
| Antibody | Anti-Bax  (rabbit polyclonal) | Immunological Sciences | Cat. No. AB-10230 | WB (1:1000) |
| Antibody | Anti- Caspase-3 (active)  (rabbit polyclonal) | Novus Biologicals | Cat. No. NB100-56113 | WB (1:1000) |
| Antibody | Anti-HO-1  (rabbit polyclonal) | Enzo Life Sciences | Cat. No. ADI-SPA-895 | WB (1:1000) |
| Antibody | Anti-GAPDH  (mouse monoclonal) | Abcam | Cat. No. ab8245 | WB (1:10000) |
| Antibody | Anti-α-tubulin  (mouse monoclonal) | Sigma-Aldrich | Cat. No. T5168 | WB (1:5000) |
| Antibody | Anti-mouse IgG,  HRP-linked | Cell Signaling | Cat. No. #7076S | WB (1:2500) |
| Antibody | Anti-rabbit IgG,  HRP-linked | Cell Signaling | Cat. No. #7074S | WB (1:2500) |
| Commercial kit | Mouse IL-1β ELISA Kit | Immunological Sciences | Cat. No. IK-4205 |  |
