# Supplementary material for: Auditory sensory deprivation induced by noise exposure exacerbates cognitive decline in a mouse model of Alzheimer’s disease
Source: eLife. 2021 Oct 26;10:e70908. doi: 10.7554/eLife.70908 (PMC8547960; doi:10.7554/eLife.70908)
Supplement: Source data 1. [file elife-70908-supp2.zip › WB Source data/Figure 10-supplement 1-Source data/Figure 10-supplement 1-source data.docx]

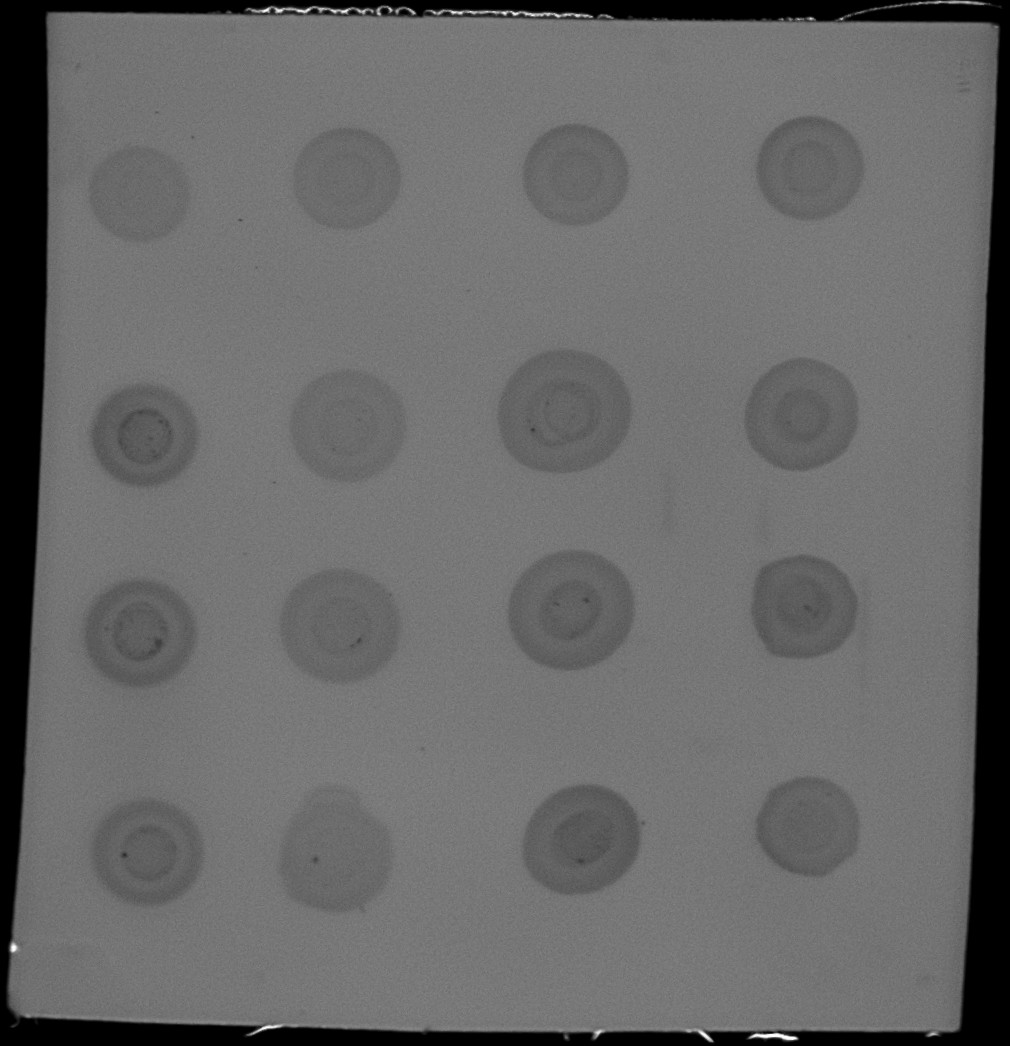


**Ponceau S**

AD

NN

NE


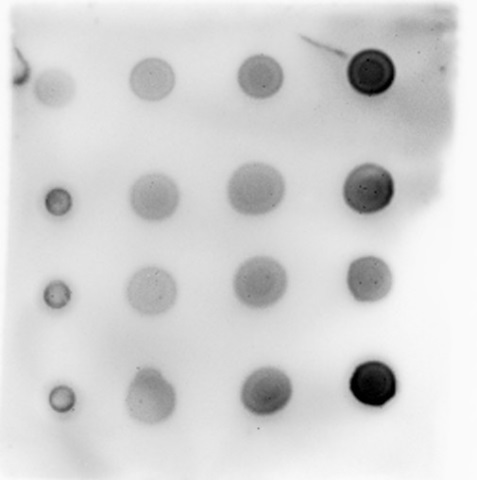


AD

NN

NE

**NT**

**Active-Caspase 3**

AD

NE

NN


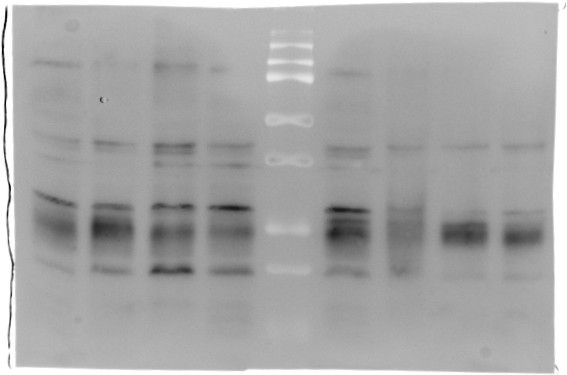


kDa

20

25

37

50

NN

NE

AD


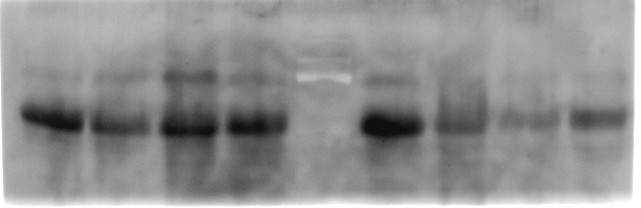


**α-Tubulin**

kDa

50

**Bax**

**GAPDH**

AD

NE

NN


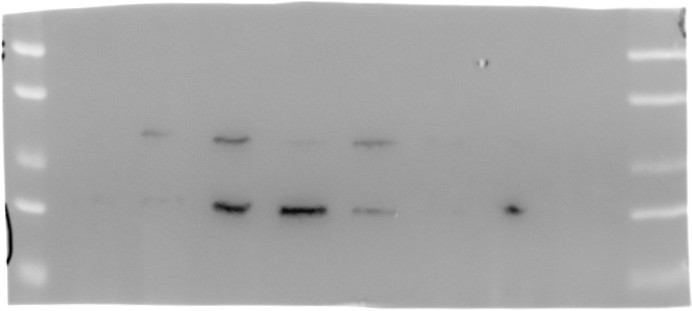


kDa

NN

NE

AD


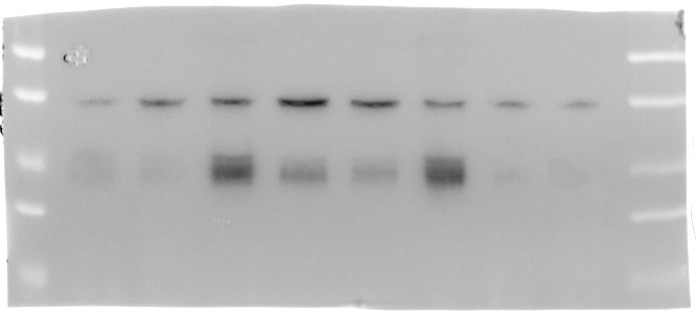


20

37

25

kDa

25

20

37

Uncropped WB from Figure 10-supplement 1
