# Supplementary material for: Auditory sensory deprivation induced by noise exposure exacerbates cognitive decline in a mouse model of Alzheimer’s disease
Source: eLife. 2021 Oct 26;10:e70908. doi: 10.7554/eLife.70908 (PMC8547960; doi:10.7554/eLife.70908)
Supplement: Source data 1. [file elife-70908-supp2.zip › WB Source data/Figure 11- Source data/Figure 11- Source data.docx]

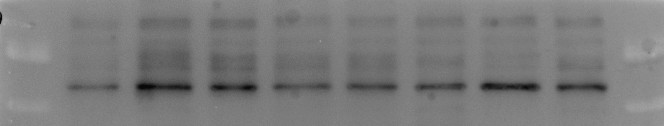
SOD2

NE

AD

WT

NN

NE

NN

WT

AD

NE

NN

NN

kDa

20

25


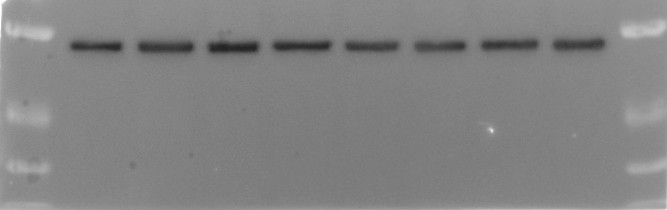


NE

kDa

25

37

GAPDH

20

NN

WT

AD

NN

NE

NE


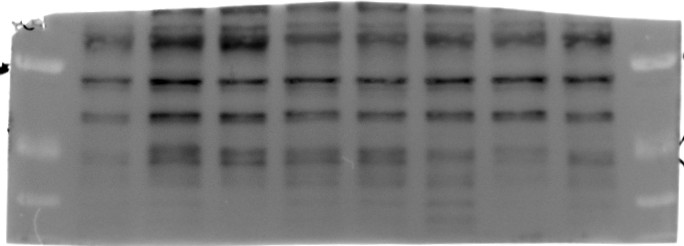


20

25

37

HO-1

Uncropped western blot from Figure 11
