# Supplementary material for: Auditory sensory deprivation induced by noise exposure exacerbates cognitive decline in a mouse model of Alzheimer’s disease
Source: eLife. 2021 Oct 26;10:e70908. doi: 10.7554/eLife.70908 (PMC8547960; doi:10.7554/eLife.70908)
Supplement: Source data 1. [file elife-70908-supp2.zip › WB Source data/Figure 8- Source data/Figure 8- Source data.docx]

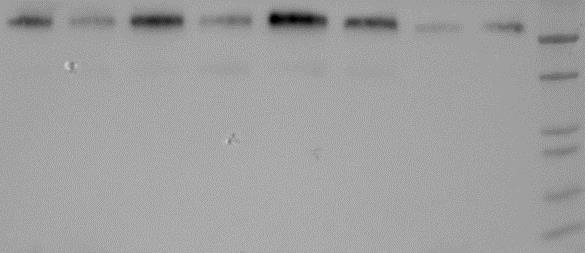
pTau^Ser396^

AD

kDa

WT

NN

NE

NN

NE

10

15

20

25

37

50

AD

WT


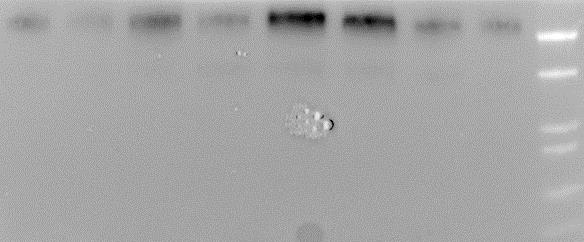


kDa

NN

NE

NN

NE

10

15

20

25

37

50

Tau


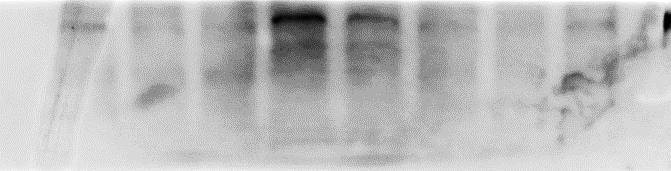
TNF-α

AD

kDa

WT

NE

NN

NN

NE

25

20

AD

NN

NE

NN

NE

WT


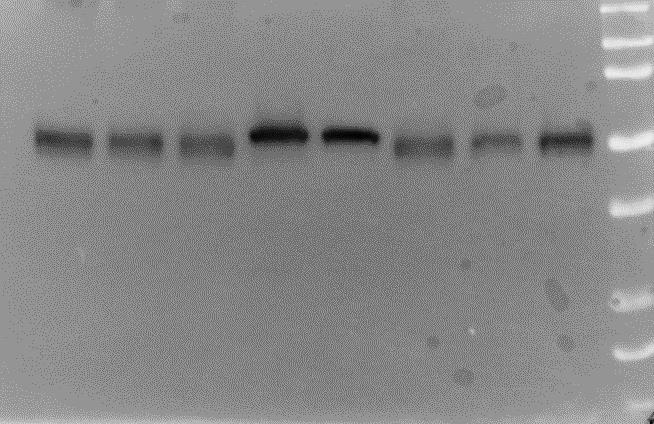


kDa

75

150

100

37

50

α-tubulin

15

25

20

Uncropped western blot from Figure 8
