# Supplementary figures and images for: Auditory sensory deprivation induced by noise exposure exacerbates cognitive decline in a mouse model of Alzheimer’s disease
Source: eLife. 2021 Oct 26;10:e70908. doi: 10.7554/eLife.70908 (PMC8547960; doi:10.7554/eLife.70908)

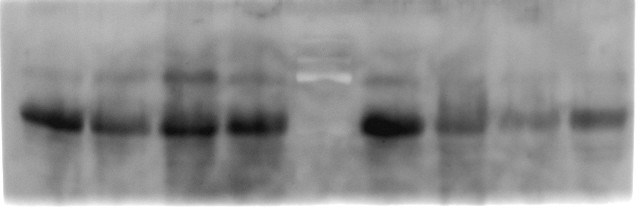

Supplement: Source data 1. [file elife-70908-supp2.zip › WB Source data/Figure 10-supplement 1-Source data/Full Image alpha-tubulin.jpg]

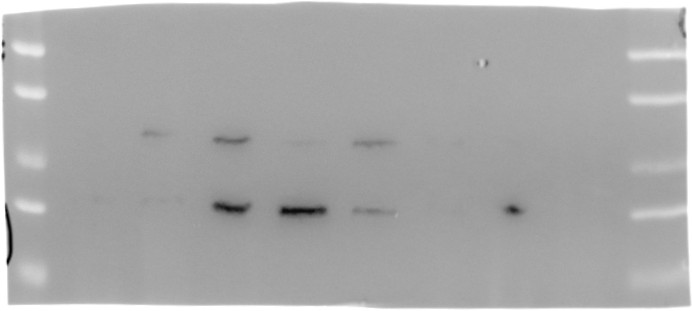

Supplement: Source data 1. [file elife-70908-supp2.zip › WB Source data/Figure 10-supplement 1-Source data/Full Image BAX.jpg]

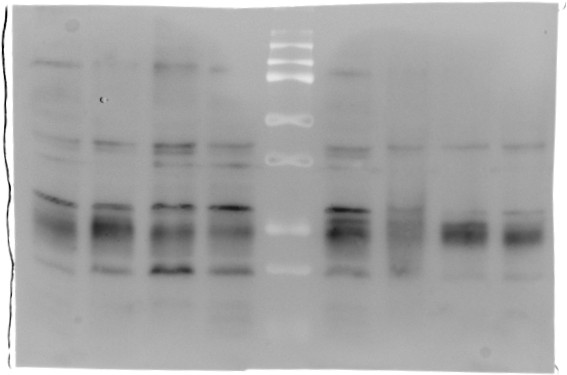

Supplement: Source data 1. [file elife-70908-supp2.zip › WB Source data/Figure 10-supplement 1-Source data/Full Image caspase-3.jpg]

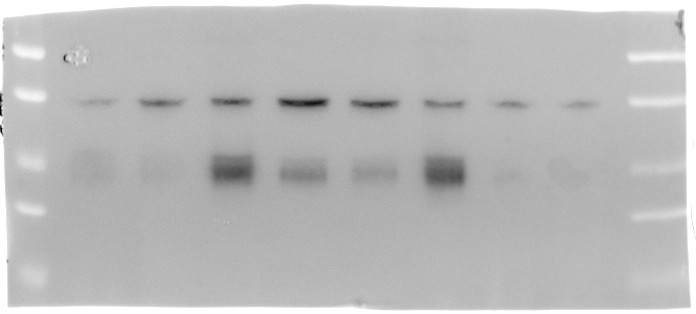

Supplement: Source data 1. [file elife-70908-supp2.zip › WB Source data/Figure 10-supplement 1-Source data/Full Image GAPDH.jpg]

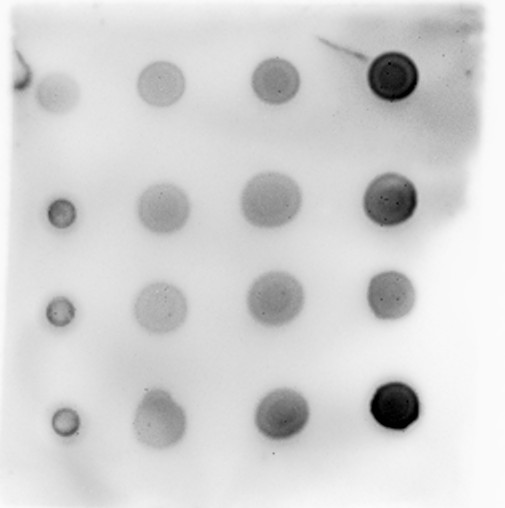

Supplement: Source data 1. [file elife-70908-supp2.zip › WB Source data/Figure 10-supplement 1-Source data/Full Image NT.jpg]

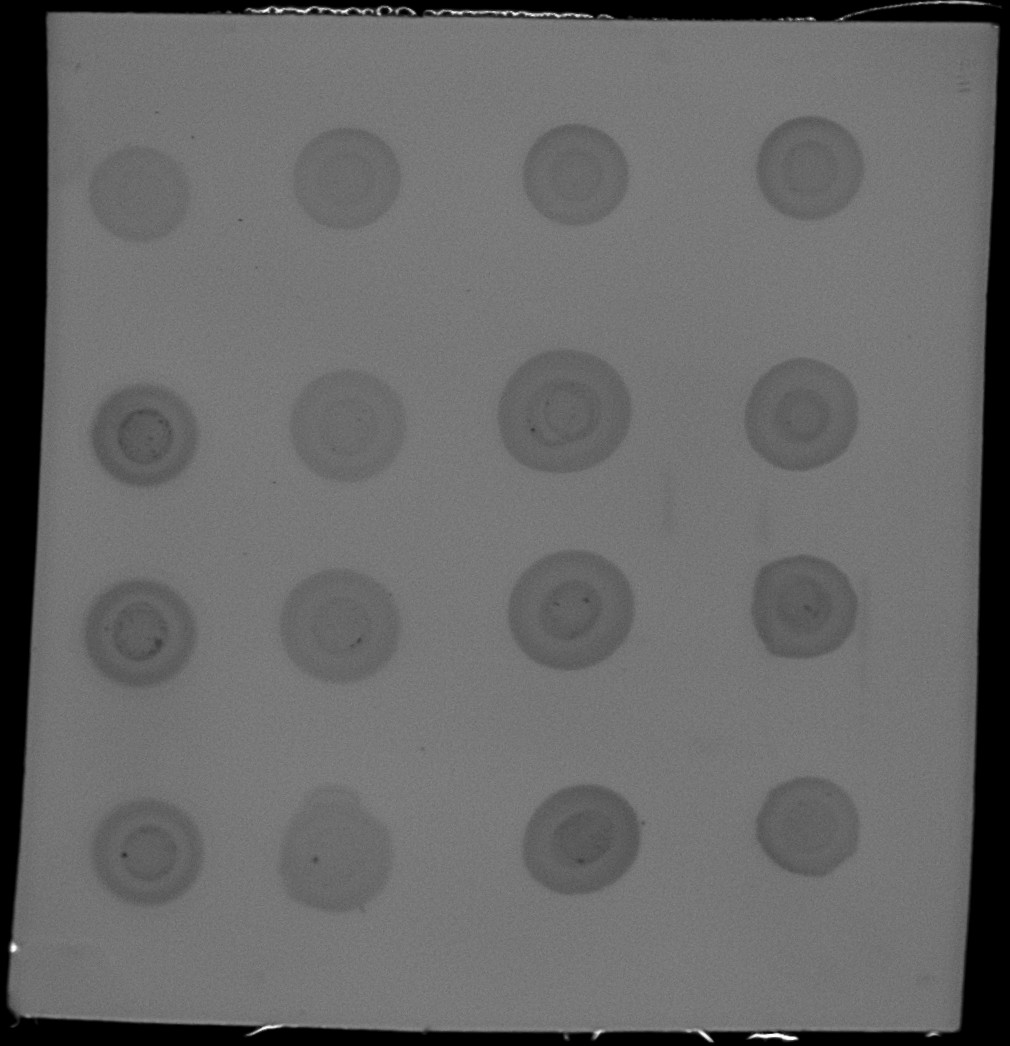

Supplement: Source data 1. [file elife-70908-supp2.zip › WB Source data/Figure 10-supplement 1-Source data/Full Image Ponceau S.jpg]

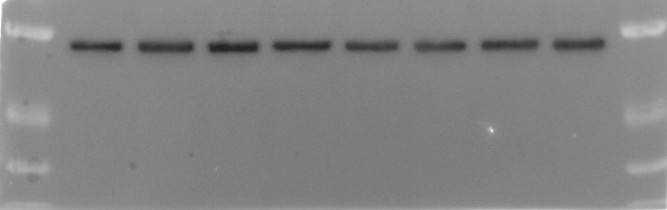

Supplement: Source data 1. [file elife-70908-supp2.zip › WB Source data/Figure 11- Source data/Full image GAPDH.jpg]

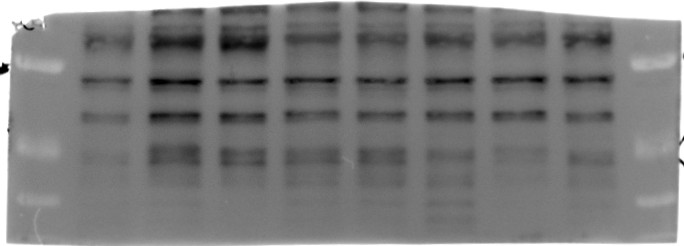

Supplement: Source data 1. [file elife-70908-supp2.zip › WB Source data/Figure 11- Source data/Full image HO-1.jpg]

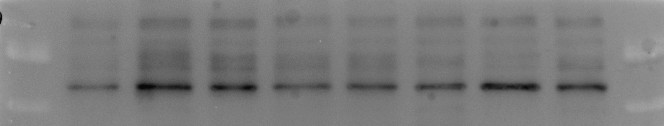

Supplement: Source data 1. [file elife-70908-supp2.zip › WB Source data/Figure 11- Source data/Full image SOD2.jpg]

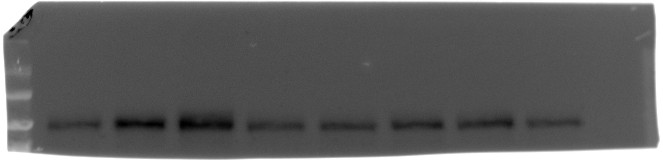


NE

NN

NE

AD

WT

NN

150

100

200

kDa


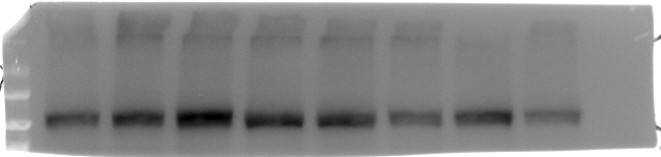


NN

NE

NE

NN

AD

WT

kDa

200

150

100

Uncropped western blot from Figure 3-supplement 2

Supplement: Source data 1. [file elife-70908-supp2.zip › WB Source data/Figure 3- supplement 2- Source data/Figure 3-supplement 2-Source data.docx]

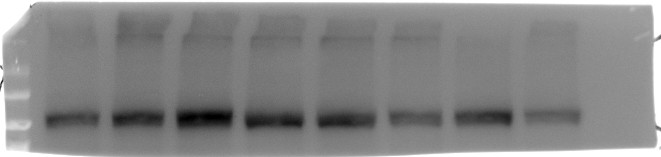

Supplement: Source data 1. [file elife-70908-supp2.zip › WB Source data/Figure 3- supplement 2- Source data/Full Image GluR1.jpg]

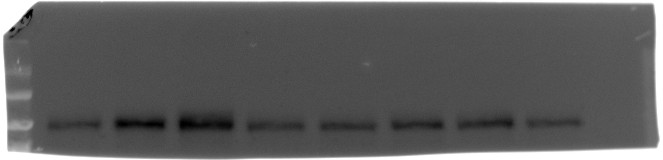

Supplement: Source data 1. [file elife-70908-supp2.zip › WB Source data/Figure 3- supplement 2- Source data/Full Image pGluA1.jpg]

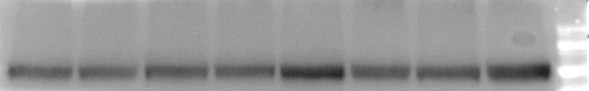

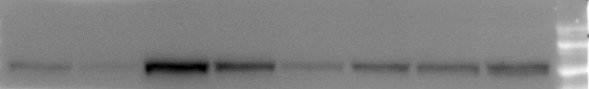


NN

NE

NN

200

150

kDa

100

kDa

200

100

150

NE

NE

AD

WT

NN

WT

AD

NE

NN

Uncropped western blot from Figure 3

Supplement: Source data 1. [file elife-70908-supp2.zip › WB Source data/Figure 3-Source data 2/Figure 3- Source data 2.docx]

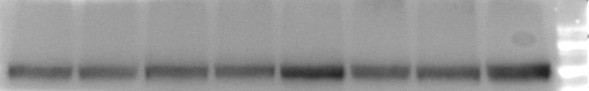

Supplement: Source data 1. [file elife-70908-supp2.zip › WB Source data/Figure 3-Source data 2/Full image GluR1.jpg]

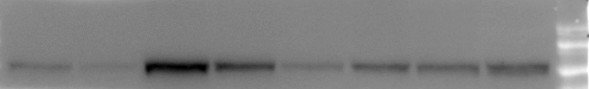

Supplement: Source data 1. [file elife-70908-supp2.zip › WB Source data/Figure 3-Source data 2/Full immage pGluR1.jpg]

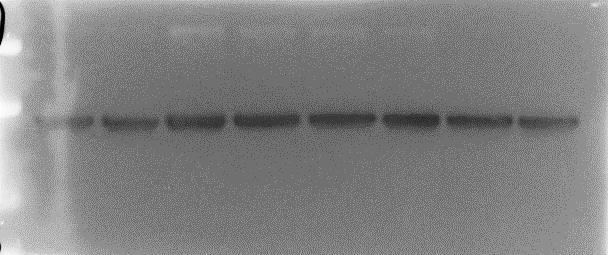

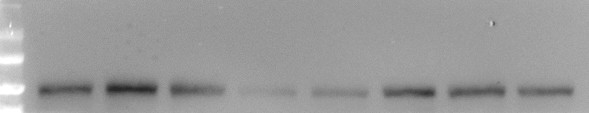


NE

AD

NE

NN

NE

AD

WT

WT

NN

NN

NE

NN

20

25

37

50

75

100

150

200

Uncropped western blot from figure 4

Supplement: Source data 1. [file elife-70908-supp2.zip › WB Source data/Figure 4-Source data 2/Figure 4- Source data 2.docx]

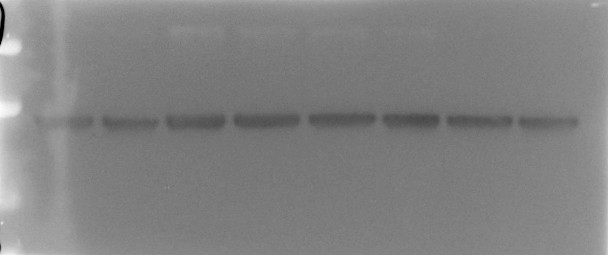

Supplement: Source data 1. [file elife-70908-supp2.zip › WB Source data/Figure 4-Source data 2/Full image GAPDH.jpg]

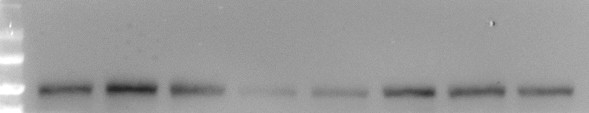

Supplement: Source data 1. [file elife-70908-supp2.zip › WB Source data/Figure 4-Source data 2/Full image PSD-95.jpg]

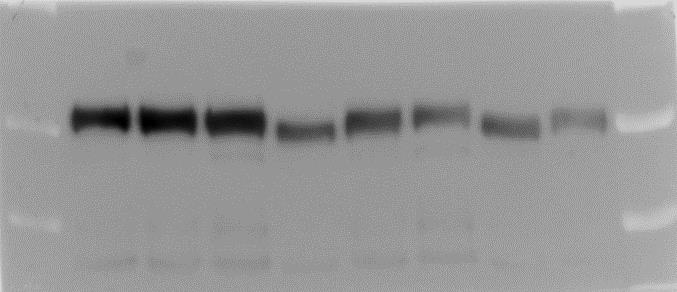

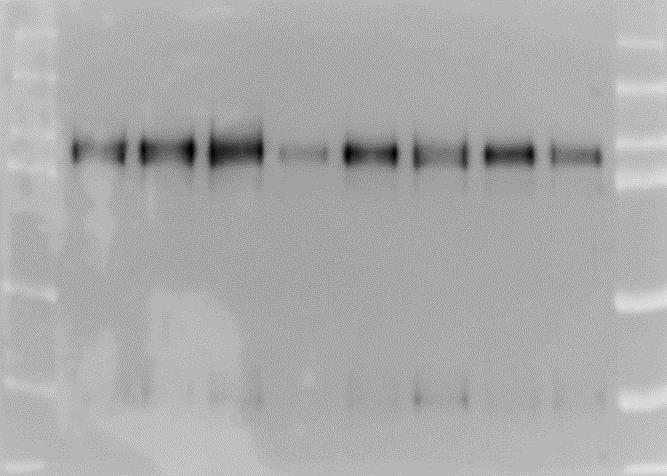


AD

AD

WT

WT

NE

NN

NE

NN

NE

NN

NE

NN

kDa

kDa

25

37

50

75

25

37

50

75

100

150

200

Uncropped WB from Figure 6

Supplement: Source data 1. [file elife-70908-supp2.zip › WB Source data/Figure 6-Source data 2/Figure 6- Source data 2.docx]

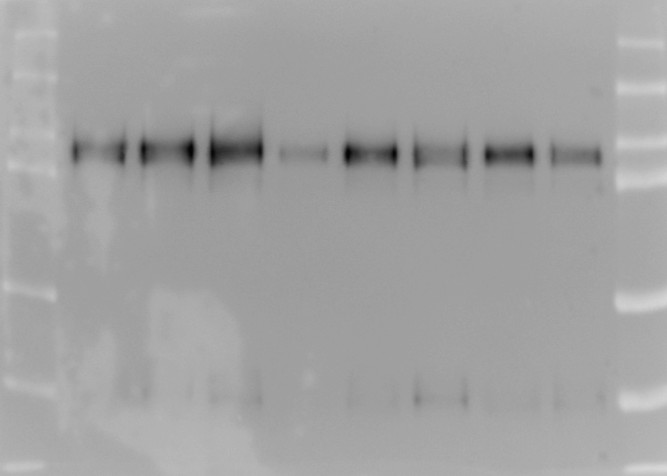

Supplement: Source data 1. [file elife-70908-supp2.zip › WB Source data/Figure 6-Source data 2/Full image PSD-95.jpg]

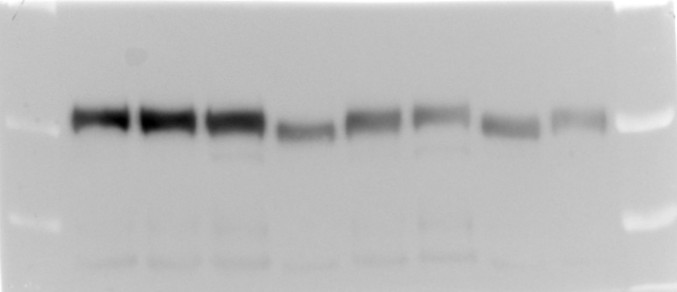

Supplement: Source data 1. [file elife-70908-supp2.zip › WB Source data/Figure 6-Source data 2/Full image Tubulin.jpg]

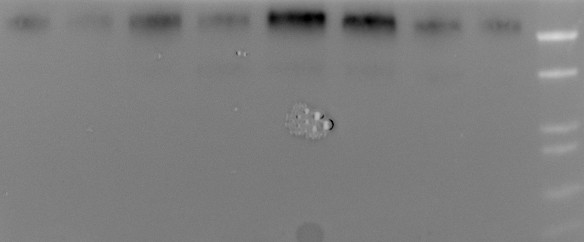

Supplement: Source data 1. [file elife-70908-supp2.zip › WB Source data/Figure 8- Source data/Ful image Tau.jpg]

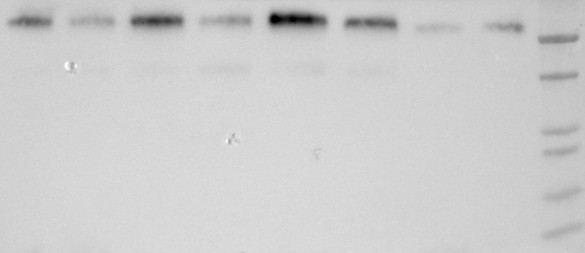

Supplement: Source data 1. [file elife-70908-supp2.zip › WB Source data/Figure 8- Source data/Full image pTauSer396.jpg]

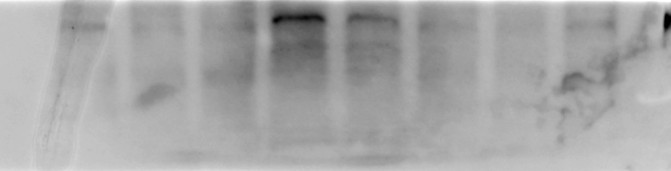

Supplement: Source data 1. [file elife-70908-supp2.zip › WB Source data/Figure 8- Source data/Full image TNF-alpha.jpg]

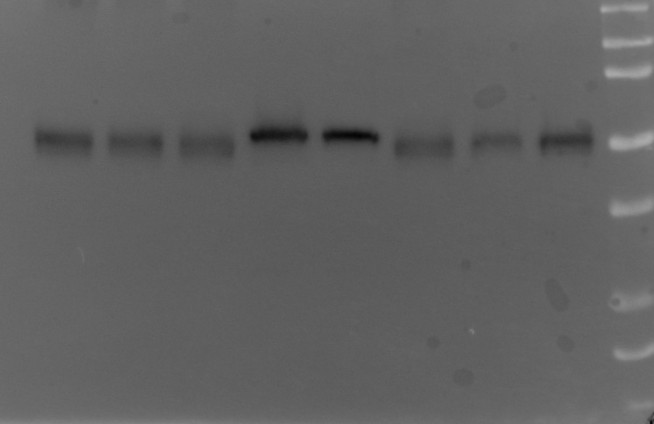

Supplement: Source data 1. [file elife-70908-supp2.zip › WB Source data/Figure 8- Source data/Full image Tubulin.jpg]
